# Creutzfeldt-Jakob disease associated with a missense mutation at codon 200 of the prion protein gene in Brazil

**DOI:** 10.1590/s1980-57642008dn10200017

**Published:** 2007

**Authors:** Jerusa Smid, Vilma Regina Martins, Michele Christine Landemberger, Daniele Riva, Renato Anghinah, Ricardo Nitrini

**Affiliations:** 1 Behavioral and Cognitive Neurology Unit, Departament of Neurology, University of São Paulo School of Medicine; 2 Ludwig Institute for Cancer Research, Hospital Alemão Oswaldo Cruz, São Paulo; 3 São Paulo Syrian-Lebanese Hospital.

**Keywords:** prion, genetic Creutzfeldt-Jakob disease, E200K

## Abstract

Genetic Creutzfeldt-Jakob disease (gCJD) represents less than 15% of CJD cases, and its clinical picture may be either indistinguishable from that of sporadic CJD (sCJD) or be atypical, usually with younger onset and longer duration. We report a case of 59-year old Brazilian man who presented rapidly progressive cognitive decline and cerebellar ataxia. EEG revealed periodic activity. A brother and a cousin of the patient had CJD. A point mutation at codon 200 (E200K) of the prion protein gene (PRNP) was found and death occurred 11 months after onset of symptoms. Autopsy was not performed. The clinical presentation of gCJD associated with E200K, which is the most frequent PRNP mutation, is quite similar to sCDJ. This is the first report of E200K mutation in Brazil, and it is possible that a more systematic search for its occurrence may show it to be relatively frequent in Brazil.

## Abstract (pt)

A forma genética da doença de Creutzfeldt-Jakob (gDCJ) representa aproximadamente 15% dos casos de DCJ e o quadro clínico pode ser indistinguível do observado na forma esporádica (eDCJ) ou pode ser atípico, geralmente com início precoce e maior sobrevida. Relatamos o caso de paciente do sexo masculino, 59 anos, que apresentou declínio cognitivo rapidamente progressivo associado a ataxia cerebelar. EEG revelou atividade periódica. DCJ havia sido diagnosticada no irmão e prima do paciente. A avaliação genética evidenciou mutação de ponto no códon 200 (E200K). Óbito ocorreu 11 meses após o início dos sintomas. Não foi realizada autópsia. O quadro clínico da gDCJ associada a E200K é semelhante ao da eDCJ, sendo esta a mutação mais freqüente no gene da proteína prion (PRNP). Este é o primeiro relato da mutação E200K no Brasil, e é possível que a pesquisa rotineira de mutações no PRNP possa identificar maior freqüência desta mutação em nosso meio.

Prion diseases, or transmissible spongiform encephalopathies, are neurodegenerative disorders and one of the causes of secondary dementia. These diseases are rare, affecting about one or two people per million per annum worldwide. These diseases are characterized by the deposition of an abnormal form of prion protein and are classified into Creutzfeldt-Jakob disease (CJD), Gerstmann-Straussler-Scheinker disease (GSS), kuru and fatal familial insomnia (FFI).^1^

The human prion diseases are classified as sporadic, inherited and acquired. The most common prion disease is CJD, which is sporadic in about 85% of the cases, while genetic form of CJD(gCJD) are responsible for more than 10% of CJD cases.^2,3^

The gCJD forms are autosomal dominantly inherited with high penetrance. There are more than 50 known pathogenic point and insertion mutations in the open reading frame of the prion protein gene (PRNP), which has 253 codons and is located in the short arm of chromosome 20.^4^ At least 13 point mutations in the PRNP have been described to cause a clinical picture that is indistinguishable from that of sporadic CJD (sCJD).^4^

Missense mutations at codons 105, 148, 178 and 183 cause atypical CJD, usually with younger onset and longer duration than sCJD, while those at codons 180, 188, 196, 200, 208, 203, 210, 211 and 232 cause a rather typical sCJD phenotype.^4^ The diagnosis of gCJD may be particularly difficult when data on the family history is missing or when there is no other case in the family, a fairly common scenario in late-onset disease.

The aim of this paper is to report a Brazilian patient with genetic CJD associated with a PRNP mutation at codon 200 (E200K).

## Case report

A 59-year old man, trader, born in Sao Paulo (Brazil) with Portuguese ancestry, presented with asthenia, pain in both legs, and apathy that had started 60 days prior to seeking our service. On initial symptoms he was seen by a psychiatrist who diagnosed a depressive episode and prescribed sertraline. There was no clinical improvement and his condition rapidly deteriorated, with the development of memory disturbances and visual hallucinations. One month after the beginning of the symptoms he was seen by one of us (DR) in the private office, when delirium with visual hallucinations and gait disturbance caused by axial ataxia were diagnosed. Routine blood tests were normal or negative. MRI showed increased diffusion signal in cerebral cortex, putamina and caudate nuclei. EEG revealed periodic sharp waves on a background of slow activity with a right parietal predominance. His condition continued to deteriorate, and two months after onset he was already bedridden, apathetic and caregivers reporting that he did not sleep during day or night, but lay with his eyes closed murmuring unintelligible utterances. Myoclonus was not observed.

He was the fifth of ten siblings, and his oldest brother had died at 58 years’ old with the clinical diagnosis of sCJD. His father had died without dementia at the age of 94, and his mother was 88 years old and has no cognitive problems. According to the caregivers, a female paternal cousin had definite diagnosis of CJD established by autopsy, in England.

The extraction of DNA from peripheral blood cells was performed following the informed consent of family members. The analysis of the complete PRNP gene open reading frame gene showed a point mutation causing substitution of glutamate by lysine in codon 200 and methionine homozigozity in codon 129.

The patient was treated with quinacrine 75 mg tablets, three times per day, with no improvement. His condition progressed to akinetic mutism, and he died 11 months after the onset of symptoms. Autopsy was not performed.

## Discussion

The phenotype of genetic forms of prion diseases can be extremely variable, including classical CJD, GSS and FFI. The definitive diagnosis is made by direct demonstration of causative mutation in PRNP, even in the absence of classical clinical features.

The most frequent mutation in PRNP is at codon 200.^5,6^ This mutation results in the substitution of glutamate (E) by lysine (K). The prevalence of the E200K mutation is especially high in Chile,^7^ Italy,^8^ Slovakia,^9^ Hungary,^10^ and Israel, particularly among Jews of Libyan and Tunisian origins.^11,12^ A study of all forms of CJD in Slovakia found that 74.2% of the cases were associated with this point mutation, differing from most countries where sCJD is more frequent.^9^ Additionally, the penetrance of the E200K mutation was around 60% within these slovanian patients.^13^ Thus, this would explain why the patient’s father who was supposed to have the mutation (a patient’s paternal cousin had a definitive diagnose of prion disease) did not develop the disease until the age of 94 when he died.

This is the first report of gCJD presenting E200K mutation in Brazil, where no epidemiological studies have been performed to investigate the proportion of sporadic, acquired or familial CJD. To date, two cases of acquired CJD associated with human growth hormone therapy have been reported,^14,15^ while only two genetic forms of CJD associated with mutation at codon 210 and at codon 183 have been described.^16,17^ The clinical presentation of fCJD associated with E200K is quite similar to sCDJ. Studies have shown similar mean age of onset, mean duration of disease, as well as similar presenting symptoms and clinical courses.^11,18-20^ The penetrance of this mutation is high, increases with age and is complete by age 85.^21,22^ Additionally the polymorphism at codon 129 is associated with susceptibility and clinical presentation of prion diseases. The presence of methionine in homozygozity or in heterozygosity seems to protect against sCJD. Conversely, in Slovakian cases of gCJD, methionine-homozygous patients at codon 129 had a shorter duration of disease (3.7±2 months) than in the heterozygous patients.^9^ Our patient was methionine-homozygous at codon 129 and had an 11-month disease course.

Considering that, as the case presented, many patients with gCJD can be clinical classified as probable CJD according to the WHO criteria^23^, it has been suggested that all cases of CJD should have PRNP gene sequenced, even in the absence of familial history.^24^ Adopting this procedure we probably would diagnose more cases of gCJD, improving our knowledge of the epidemiology of prion diseases in Brazil.

The Jews of Libyan and Tunisian origins, and the Spanish, Italian and Chilean non-Jewish populations all share a major haplotype, suggesting that the E200K mutation originated from a single mutation that occurred in Spain before the expulsion of the Sephardic Jews.^6^ It is possible that Brazilian population also has this haplotype, as Brazil received part of this migrating Sephardic Jews and Spanish population who came to South America. According to this hypothesis, it would be reasonable to expect the frequency of this mutation to be relatively high in our population.

## References

[1] Prusiner SB (1991). Molecular biology of prion diseases. Science.

[2] Masters CL, Gajdusek DC, Gibbs CJ (1981). The familial occurrence of Creutzfeldt-Jakob disease and
Alzheimer's disease. Brain.

[3] Prusiner SB, Hsiao KK (1994). Human prion diseases. Ann Neurol.

[4] Kong Q, Surewicz WK, Petersen RB, Prusiner SD, Petersen RB (2004). Inherited prion diseases. Prion Biology and Diseases.

[5] Knight SG, Will RG (2004). Prion diseases. J Neurol Neurosurg Psychiatry.

[6] Lee HS, Sambuughin N, Cervenakova L (1999). Ancestral origins and worldwide distribution of the PRNP 200K
mutation causing familial Creutzfeldt-Jakob disease. Am J Hum Genet.

[7] Brown P, Galvez S, Goldfarb LG (1992). Familial Creutzfeldt-Jakob disease in Chile is associated with
the codon 200 mutation of the PRNP amyloid precursor gene on chromosome
20. J Neurol Sci.

[8] Ladogana A, Puopolo M, Poleggi A (2005). High incidence of genetic human transmissible spongiform
encephalopathies in Italy. Neurology.

[9] Mitrova E, Belay G (2002). Creutzfeldt-Jakob disease with E200K mutation in Slovakia
characterization and development. Acta Virol.

[10] Kovacs GG, Majtenyi K (2005). Creutzfeldt-Jakob disease in Hungary. Folia Neuropathol.

[11] Meiner Z, Gabizon R, Prusiner SB (1997). Familial Creutzfeldt-Jakob disease Codon 200 prion disease in
Libyan Jews. Medicine (Baltimore).

[12] Hsiao K, Meiner Z, Kahana E (1991). Mutation of the prion protein in Libyan Jews with
Creutzfeldt-Jakob disease. N Engl J Med.

[13] Mitrova E, Belay G (2002). Creutzfeldt-Jakob disease with E200K mutation in Slovakia
characterization and development. Acta Virol.

[14] Caboclo LO, Huang N, Lepski GA (2002). Iatrogenic Creutzfeldt-Jakob disease following human growth
hormone therapy case report. Arq Neuropsiquiatr.

[15] Macario ME, Vaisman M, Buescu A (1991). Pituitary growth hormone and Creutzfeldt-Jakob
disease. Br Med J.

[16] Nitrini R, Rosemberg S, Passos-Bueno MR (1997). Familial spongiform encephalopathy associated with a novel prion
protein gene mutation. Ann Neurol.

[17] Huang N, Marie SK, Kok F, Nitrini R (2001). Familial Creutzfeldt-Jakob disease associated with a point
mutation at codon 210 of prion protein gene. Arq Neuropsiquiatr.

[18] Brown P, Cathala F, Castaigne P, Gajdusek DC (1986). Creutzfeldt-Jakob disease Clinical analysis of a consecutive
series of 230 neuropathologically verified cases. Ann Neurol.

[19] Brown P, Goldfarb LG, Gibbs CJ Jr, Gajdusek DC (1991). The phenotypic expression of different mutations in transmissible
familial Creutzfeldt-Jakob disease. Eur J Epidemiol.

[20] Kahana E, Zilber N, Abraham M (1991). Do Creutzfeldt-Jakob disease patients of Jewish Libyan origin
have unique clinical features. Neurology.

[21] Chapman J, Ben-Israel J, Goldhammer Y, Korczyn AD (1994). The risk of developing Creutzfeldt-Jakob disease in subjects with
the PRNP gene codon 200 point mutation. Neurology.

[22] Spudich S, Mastrianni JA, Wrensch M (1995). Complete penetrance of Creutzfeldt-Jakob disease in Libyan Jews
carrying the E200K mutation in the prion protein gene. Mol Med.

[23] WHO recommended surveillance standards.

[24] Kovacs GG, Puopolo M, Ladogana A (2005). Genetic prion disease the EUROCJD experience. Hum Genet.

